# Beyond human ears: navigating the uncharted risks of AI scribes in clinical practice

**DOI:** 10.1038/s41746-025-01895-6

**Published:** 2025-09-24

**Authors:** Maxim Topaz, Laura Maria Peltonen, Zhihong Zhang

**Affiliations:** 1 Columbia University School of Nursing, New York, NY USA; 2 Columbia University Data Science Institute, New York, NY USA; 3 Center for Home Care Policy & Research, Visiting Nurse Service of New York, New York, NY USA; 4 Department of Health and Social Management, University of Eastern Finland, Wellbeing Services County of North Savo, Kuopio, Finland

**Keywords:** Health care, Medical research

## Abstract

Artificial intelligence (AI) scribes have been rapidly adopted across health systems, driven by their promise to ease the documentation burden and reduce clinician burnout. While early evidence shows efficiency gains, this commentary cautions that adoption is outpacing validation and oversight. Without greater scrutiny, the rush to deploy AI scribes may compromise patient safety, clinical integrity, and provider autonomy.

Artificial intelligence (AI) scribes, tools that passively capture and summarize clinical conversations, are rapidly reshaping medical documentation. Promising to reduce the administrative burden that contributes to clinician burnout, these tools are now used by approximately 30% of physician practices, with adoption growing across major health systems and electronic health records (EHR) platforms^1^. Studies suggest that AI scribes can reduce documentation time by 20% to 30%, offering the potential to improve clinician well-being and expand capacity for patient care^2,3^. Yet the speed of adoption has outpaced validation, transparency, and regulatory oversight. To appreciate the unique risks of AI scribes, it is helpful to contrast them briefly with traditional human scribes and automated dictation tools. Human medical scribes manually document encounters in real-time and, in randomized trials, are more than four times as likely to produce notes physicians rate as ‘accurate’ compared with standard self-documentation^4^. Automated speech-recognition dictation systems generally have higher error rates—typically 7–11%—owing to the complexity of medical jargon and accent variability^5^. Modern ambient AI scribes leveraging large language models report lower overall error rates (≈1–3%) but introduce distinct failure modes such as AI hallucinations (AI-generated content that appears plausible but has no basis in reality), critical omissions, misattribution, and contextual misinterpretations, creating new safety challenges^6,7^.

This comment examines the tension between the potential benefits of AI scribes in reducing documentation burden and the substantial risks associated with their premature deployment. We argue that without greater scrutiny, we risk compromising patient safety, clinical integrity, and provider autonomy in our rush to implement technological solutions to healthcare’s administrative challenges.

## The rapid rise of ambient documentation

The scale of implementation is striking. One large healthcare system reported over 7000 physicians utilizing AI scribes in more than 2.5 million patient encounters over just 14 months^8^. Major EHR vendors have integrated ambient documentation capabilities directly into their platforms, further accelerating adoption. AI scribes vary significantly in design and implementation. Some operate as standalone third-party applications requiring manual transfer of content into EHR systems, while others integrate directly into major platforms for seamless workflow integration. Systems also differ in processing approaches, with some offering real-time ambient listening versus post-encounter processing of recorded conversations.

This rapid uptake of AI scribes is primarily driven by the promise of substantial efficiency gains across healthcare disciplines. While most implementations have focused on physicians, nurses and other healthcare professionals who dedicate significant time to documentation tasks could potentially benefit from similar efficiency gains. In a quality improvement study of 45 clinicians from 17 specialties, including nurse practitioners and physician assistants, ambient AI scribes reduced documentation time by a median of 2.6 min per appointment and cut after-hours EHR work by 29.3%^9^. Similarly, an observational study involving 119 allied health professionals (e.g., physiotherapists, podiatrists, and occupational therapists) found a 33% reduction in documentation time, along with increased productivity and satisfaction, without affecting patient experience^10^. These time savings offer a meaningful opportunity to improve clinician well-being and expand capacity for direct patient care^11,12^.

## Navigating the risks: accuracy, errors, and the “Black Box” concern

Despite these promising benefits, significant concerns exist regarding the accuracy and reliability of AI-generated clinical notes. Even studies reporting relatively low AI hallucination rates (around 1–3%) acknowledge that in healthcare, even a small percentage of errors can have profound implications for patient safety^6^. However, these reported rates vary significantly based on evaluation methodologies, with some studies defining AI hallucinations narrowly as factual inaccuracies while others include broader categories of clinical inconsistencies and omissions. Real-world experiences have illustrated multiple types of documentation failures:

- **AI hallucinations (Fabrications):** AI systems can generate entirely fictitious content, such as documenting examinations that never occurred or creating nonexistent diagnoses^7^.
- **Omissions:** Critical information discussed during encounters may be absent from the generated note, including symptoms, concerns, or assessment findings^7^.
- **Misinterpretations:** Context-dependent statements can be misconstrued, leading to incorrect documentation of treatments, medications, or care plans^7^. Beyond these audio-based errors, AI scribes are fundamentally limited to audio input and cannot capture nonverbal communication, such as patient body language or visual signs of distress that human scribes might observe. Human scribes from the same communities may also better recognize and document social determinants of health and cultural context that AI systems might overlook, representing a loss of valuable contextual information that extends beyond verbal communication.
- **Speaker Attribution Errors:** Current systems struggle to consistently distinguish between multiple speakers, potentially attributing patient statements to clinicians or vice versa^7^. Additionally, speech recognition systems underlying AI scribes exhibit systematic performance disparities that result in significantly higher error rates for African American speakers and reduced accuracy when transcribing speech from African American patients compared to White patients^13,14^. These disparities reflect the limitations of training data and algorithmic design choices that disproportionately affect certain linguistic patterns and accents.

These risks are not new. Earlier speech recognition systems have caused patient harm due to transcription errors—such as erroneously documenting “no vascular flow” instead of “normal vascular flow,” prompting an unnecessary procedure, or confusing the location of a tumor, resulting in surgery on the wrong site^5^. These historical failures parallel the AI hallucinations now seen in advanced AI scribes, suggesting that core risks persist despite technological advancement. Compounding the issue is the “black box” nature of these systems. The underlying neural network algorithms are not constrained by established medical knowledge, making it difficult to understand how they arrive at specific conclusions or predict when errors might occur^7^. This lack of transparency makes it challenging to identify potential biases within the system and ultimately ensure the reliability of generated documentation. Emerging explainability techniques, such as attention visualization (which highlights which parts of conversations most influenced specific documentation decisions) and SHapley Additive exPlanations (SHAP) frameworks (which identify key linguistic features that trigger certain AI outputs), offer promising approaches to enhance AI transparency; however, their effectiveness and practical implementation for clinical documentation systems require further validation.

## The Documentation Gap and AI Scribes

Research has already identified significant gaps between verbal communication in healthcare settings and what gets documented in EHR. A study by Song et al. found that approximately 50% of patient problems and 21% of interventions discussed during verbal communication between nurses and patients in home healthcare were never documented in the EHR^15^. These gaps occurred for various reasons, including problems being outside the scope of practice for the conversing clinicians or issues not deemed severe enough to warrant documentation. This raises critical questions about how AI scribes might change documentation patterns. Will these systems document everything discussed, potentially creating information overload? Or will they selectively filter information based on unclear criteria? Either approach presents challenges. Comprehensive documentation might capture previously missed information but could also clutter the medical record with less clinically relevant details.

Conversely, if AI scribes apply filtering algorithms, they might perpetuate or even exacerbate existing documentation gaps without the contextual understanding that human clinicians possess. These risks may disproportionately affect vulnerable populations who are less able to engage in effective self-advocacy. Our recent research documented significant disparities in automatic speech recognition performance, with AI systems showing reduced accuracy when transcribing speech from Black patients compared to White patients^14^. Such disparities suggest that patients with non-standard accents, limited English proficiency, or those from marginalized communities may receive inadequate documentation of their concerns, potentially missing critical clinical information that could affect their care. The implications for interprofessional communication are significant. If different care team members use AI scribes with varying algorithms or sensitivities, documentation inconsistencies could widen communication gaps—undermining, rather than enhancing, coordinated care.

Research consistently demonstrates that physicians face significant information overload in EHR, with studies linking excessive clinical data to increased stress levels and documentation burden^16^. AI scribes may worsen this paradox by comprehensively documenting all discussions, potentially overwhelming providers who must review and validate extensive AI-generated content. Given that our previous research found approximately 50% of patient problems discussed verbally were never documented in EHRs^15^, AI scribes that capture everything may create an inverse problem of information excess rather than addressing the original issue of selective documentation gaps.

Further compounding these concerns, recent evidence suggests AI scribes may create unintended consequences that paradoxically increase physician workload. A study found AI scribes saved only 34 s per note with significant individual variability, meaning many physicians experienced minimal benefit^17^. Healthcare organizations may respond by increasing patient volume expectations based on promised efficiency gains, creating a workload paradox where modest time savings are offset by greater demands and the cognitive burden of reviewing AI-generated errors.

## Ethical, transparency, and legal considerations

The integration of AI scribes into healthcare raises foundational questions about patient consent. While consent for recording clinical conversations is essential, legal requirements vary across jurisdictions^3^. Healthcare organizations must prioritize Health Insurance Portability and Accountability Act (HIPAA) compliance, implement robust data encryption, and establish secure storage protocols. Beyond immediate consent for recording, AI scribes raise complex questions about secondary data use that patients may not anticipate when consenting to clinical documentation. The vast repositories of patient conversations generated by these systems create valuable datasets for AI development and research, yet patients providing clinical information to address specific health problems may not expect their data to be used for algorithm training or commercial AI development. This unconsented secondary use risks eroding patient trust, particularly among communities with historical experiences of medical exploitation. The challenge is compounded when aggregated patient data from AI scribes is used to develop new AI products or sold to third parties, creating economic value from patient interactions without explicit consent for such commercialization.

Transparency regarding how the technology works, the reasons for its use, and the measures taken to protect patient privacy are paramount for building trust. Some vendors, like Amazon Web Services HealthScribe^18^, incorporate traceable transcript references to enhance verifiability, but more comprehensive transparency standards are needed.

Liability in the event of errors is another unresolved issue. Clinicians may hesitate to adopt AI scribes if they risk being held responsible for algorithm-driven documentation errors. Professional organizations, such as the Royal Australian College of Surgeons, have called for updates to civil liability frameworks to clarify accountability for harms resulting from the use of AI use in clinical care^19^. Currently, most AI scribes operate without specific FDA oversight, as they are often classified as administrative tools rather than medical devices, creating a regulatory gap that leaves safety and efficacy standards largely unaddressed. Major commercial AI scribes are marketed as HIPAA-eligible services rather than medical devices, allowing them to bypass formal FDA evaluation processes despite their direct impact on clinical documentation.

AI scribes also raise concerns about clinician autonomy, as clinicians may become overly dependent on AI-generated documentation, potentially compromising their professional judgment and independence in clinical decision-making. The reliance on algorithmic outputs may subtly shift clinical practice patterns and reduce clinicians’ control over their documentation processes.

The ethical considerations surrounding AI scribes extend beyond technical privacy concerns to fundamental questions about clinical practice and patient care. Recent literature has examined whether AI and human scribes are ethically equivalent, applying bioethical principles of beneficence, non-maleficence, autonomy, and justice to the implementation of AI scribes^20^. While AI scribes may enhance efficiency and standardize care thereby benefitting patients (beneficence), they also introduce risks of documentation errors requiring physician correction to prevent harm (maleficence), raise concerns about patient autonomy regarding consent for AI use, and may promote injustice by presenting disproportionate risks to the least advantaged patients, particularly those with non-standard accents or limited English proficiency (justice). Unlike human scribes who develop an understanding of provider preferences and can exercise judgment, AI systems operate without contextual reasoning, fundamentally altering the nature of clinical documentation.

## Critical risks and necessary safeguards

To ensure the responsible implementation of AI scribes, several key risks must be addressed with appropriate safeguards (Table 1).

**Table 1:** Key risk categories and recommended safeguards for AI scribe implementation

Risk Category	Examples of Potential Harm	Recommended Safeguards
Clinical Accuracy	AI Hallucinations (fabricated exams), Omissions of symptoms	Mandatory accuracy standards, Independent validation studies
Patient Privacy	Unauthorized recording, Data repurposing for AI training	Explicit consent protocols, Audit trails for data access
Legal Liability	Unclear responsibility for AI errors, Documentation discrepancies	Updated liability frameworks, Clear error attribution processes
Transparency	“Black box” algorithms, Proprietary systems	Required explainability standards, Open audit of error rates
Interprofessional Communication	Inconsistent documentation across care team, Widened information gaps	Team-based implementation protocols, Shared responsibility models

## The way forward: a call for balanced implementation

Integrating AI scribes represents a significant opportunity to address the documentation burden, but their rapid adoption without comprehensive validation raises substantial concerns. The following key actions are recommended:

- **Establish Rigorous Validation Standards:** Independent evaluation using standardized metrics for note accuracy, completeness, and time savings should be required before widespread implementation^17^.
- **Mandate Transparency:** Vendors should be required to disclose how their systems function, their limitations, and potential biases, including regular reporting of error rates.
- **Develop Clear Regulatory Frameworks:** Updated guidelines should define responsibility and accountability when errors occur, protecting both patients and clinicians.
- **Implement Thoughtful Clinical Protocols:** Healthcare organizations should develop robust training programs, quality assurance processes, and patient consent protocols before deploying these technologies, as demonstrated by large-scale implementations^8^. Training programs should specifically address how clinicians can effectively audit AI-generated content, including recognizing common error patterns, verifying techniques for AI-generated claims, and adopting systematic approaches to editing while maintaining clinical accuracy.
- **Invest in Research:** Dedicated funding should support independent research investigating the long-term impacts of these systems on documentation quality, clinical decision-making, and interprofessional communication, including discipline and specialty-specific evaluations^11,12^.

Implementing these recommendations requires coordinated action across multiple levels. At the federal level, regulatory agencies like the FDA must develop clear guidelines for AI scribe classification and oversight. State and local health departments should establish implementation standards that reflect regional healthcare needs. At the institutional level, hospitals and health systems must develop internal governance structures for AI scribe deployment. Different stakeholders have distinct roles: physicians and nurses should participate in validation studies and provide feedback on their clinical utility; hospital administrators must ensure adequate training and quality assurance; technology vendors should prioritize transparency and bias mitigation; patients should be informed participants in the consent process; and patient councils or community engagement exercises should be utilized to identify concerns that healthcare professionals might overlook. This multi-level, multi-stakeholder approach is essential for the responsible implementation of AI.

## Conclusion

AI scribes offer great potential to reduce clinician burnout by easing documentation burdens. However, this promise must be weighed against risks such as documentation errors, privacy concerns, and lack of transparency. Moving forward, we must balance innovation with safeguards through rigorous validation, transparency, clear regulations, and thoughtful implementation to protect patient safety and uphold clinical integrity. The key question is not whether to adopt these tools but how to do so responsibly, ensuring they enhance care without eroding trust.

## Data Availability

No datasets were generated or analyzed during the current study.

## References

[1] Tierney, A. A. et al. Ambient Artificial Intelligence Scribes: Learnings after 1 Year and over 2.5 Million Uses. *NEJM Catal*. **6**, 10.1056/CAT.25.0040 (2025).

[2] Stults, C. D. et al. Evaluation of an ambient artificial intelligence documentation platform for clinicians. *JAMA Netw. Open* **8**, e258614 (2025). 40314951 10.1001/jamanetworkopen.2025.8614 PMC12048851

[3] Albrecht, M. Enhancing clinical documentation with ambient artificial intelligence: a quality improvement survey assessing clinician perspectives on work burden, burnout, and job satisfaction. *JAMIA Open* **8**, ooaf013 (2024). 10.1093/jamiaopen/ooaf013 PMC11843214 39991073

[4] Gidwani, R. et al. Impact of scribes on physician satisfaction, patient satisfaction, and charting efficiency: A randomized controlled trial. *Ann. Fam. Med.* **15**, 427–433 (2017). 28893812 10.1370/afm.2122 PMC5593725

[5] Topaz, M. et al. Medical malpractice trends: errors in automated speech recognition. *J. Med Syst.* **42**, 153 (2018). 29987660 10.1007/s10916-018-1011-9

[6] Asgari, E. et al. A framework to assess clinical safety and hallucination rates of LLMs for medical text summarisation. *NPJ Digit Med* **8**, 274 (2025). 40360677 10.1038/s41746-025-01670-7 PMC12075489

[7] Mess, S. A., Mackey, A. J. & Yarowsky, D. E. Artificial intelligence scribe and large language model technology in healthcare documentation: advantages, limitations, and recommendations. *Plast. Reconstr. Surg. Glob. Open* **13**, e6450 (2025). 39823022 10.1097/GOX.0000000000006450 PMC11737491

[8] Tierney, A. A. et al. Ambient artificial intelligence scribes to alleviate the burden of clinical documentation. *NEJM Catal.* **5**, 10.1056/CAT.23.0404 (2024).

[9] Duggan, M. J. et al. Clinician experiences with ambient scribe technology to assist with documentation burden and efficiency. *JAMA Netw. Open* **8**, e2460637 (2025). 39969880 10.1001/jamanetworkopen.2024.60637 PMC11840636

[10] Evans, K. et al. Impact of using an AI scribe on clinical documentation and clinician-patient interactions in allied health private practice: perspectives of clinicians and patients. *Musculoskelet. Sci. Pr.* **78**, 103333 (2025). 10.1016/j.msksp.2025.103333 40328004

[11] Shah, S. J. et al. Physician perspectives on ambient AI scribes. *JAMA Netw. Open* **8**, e251904 (2025). 40126477 10.1001/jamanetworkopen.2025.1904 PMC11933996

[12] Cao, D. Y., Silkey, J. R., Decker, M. C. & Wanat, K. A. Artificial intelligence-driven digital scribes in clinical documentation: Pilot study assessing the impact on dermatologist workflow and patient encounters. *JAAD Int* **15**, 149–151 (2024). 38571698 10.1016/j.jdin.2024.02.009 PMC10988030

[13] Martin, J. L. & Wright, K. E. Bias in automatic speech recognition: the case of African American Language. *Appl. Linguist* **44**, 613–630 (2023).

[14] Zolnoori, M. et al. Decoding disparities: evaluating automatic speech recognition system performance in transcribing Black and White patient verbal communication with nurses in home healthcare. *JAMIA Open* **7**, ooae130 (2024). 39659993 10.1093/jamiaopen/ooae130 PMC11631515

[15] Song, J. et al. Do nurses document all discussions of patient problems and nursing interventions in the electronic health record? A pilot study in home healthcare. *JAMIA Open* **5**, ooac034 (2022). 35663115 10.1093/jamiaopen/ooac034 PMC9154272

[16] Nijor, S., Rallis, G., Lad, N. & Gokcen, E. Patient safety issues from information overload in electronic medical records. *J. Patient Saf.* **18**, e999–e1003 (2022). 35985047 10.1097/PTS.0000000000001002 PMC9422765

[17] Ma, S. P. et al. Ambient artificial intelligence scribes: utilization and impact on documentation time. *J. Am. Med. Inform. Assoc.* **32**, 381–385 (2025). 39688515 10.1093/jamia/ocae304 PMC11756633

[18] Amazon Web Services. Generate clinical notes with AI — AWS HealthScribe. https://aws.amazon.com/healthscribe/.

[19] MinterEllison. *AI and Healthcare: Summary of Commonwealth Consultations*. https://www.minterellison.com/articles/ai-and-healthcare-summary-of-commonwealth-consultations (2025).

[20] Klufas, T. et al. Implications of artificial intelligence scribe usage. *J. Am. Acad. Dermatol*10.1016/j.jaad.2025.04.014 (2025). 40216198 10.1016/j.jaad.2025.04.014

